# COL6A6 Peptide Vaccine Alleviates Atherosclerosis through Inducing Immune Response and Regulating Lipid Metabolism in *Apoe^−/−^* Mice

**DOI:** 10.3390/cells13181589

**Published:** 2024-09-21

**Authors:** Dongmei Tang, Yan Liu, Rui Duan, Run Lin, Zhonghao Li, Xianyan Liu, Jingrong Huang, Ming Zhao

**Affiliations:** Department of Pathophysiology, Key Lab for Shock and Microcirculation Research of Guangdong, School of Basic Medical Sciences, Southern Medical University, Guangzhou 510515, China; tang961129@126.com (D.T.);

**Keywords:** Pep_A6 vaccine, atherosclerosis, antigen-specific antibody, Th/Treg cell, lipid metabolism

## Abstract

Atherosclerosis is an autoimmune disease characterized by lipid imbalances and chronic inflammation within blood vessels, with limited preventive and treatment options currently available. In this study, a vaccine prepared with COL6A6 peptide (named the Pep_A6 vaccine) was administered to immunize *Apoe^−/−^* mice, and the immune mechanism of the Pep_A6 vaccine against atherosclerosis was first investigated. The results of arterial oil red O staining demonstrated that the Pep_A6 vaccine significantly reduced the atherosclerotic plaque area in *Apoe^−/−^* mice fed with a high-fat diet for 20 weeks. A flow cytometry analysis revealed that the Pep_A6 vaccine inhibited Th1 cell differentiation and increased the proportion of Treg cells. Furthermore, there was a significant increase in Ly6C^low^ monocytes observed in the vaccinated group. The ELISA results showed that the Pep_A6 vaccine induced a significant expression of Pep_A6-specific antibody IgG and IgG1 in mouse serum. Additionally, we found that the Pep_A6 vaccine significantly decreased serum LDL-C content and regulated the expression of genes related to liver lipid metabolism. Together, our findings suggest that the Pep_A6 vaccine alleviates atherosclerosis by inducing a positive immune response and regulating lipid metabolism, providing new insights into potential prevention strategies for atherosclerosis as an innovative vaccine.

## 1. Introduction

The pathogenesis of atherosclerosis is a multifaceted process influenced by the interplay of hyperlipidemia, chronic inflammation, and other intricate factors. Prolonged high-fat diet (HFD) consumption has been linked to severe health complications affecting various human organs and tissues [1]. This dietary pattern often leads to hyperlipidemia, characterized by aberrant blood lipid profiles, including elevated total cholesterol (CHO), triglycerides (TG), and low-density lipoprotein cholesterol (LDL-C), along with reduced levels of high-density lipoprotein cholesterol (HDL-C) [2]. Hyperlipidemia promotes the accumulation of plasma-derived lipoproteins at the endothelial injury site of arterial wall endothelium, subsequently contributing to atherosclerosis, coronary heart disease, diabetes, and other diseases [3].

Atherosclerosis is frequently the underlying cause of cardiovascular events, representing a chronic inflammatory disorder [4]. The current evidence suggests that both adaptive and innate immunity mechanisms play pivotal roles in driving chronic inflammation within atherosclerotic lesions [5], and the differentiation of several T cell types and monocytes significantly impacts the progression of this disease. T cells have been documented in all stages of atherosclerosis and can either activate or suppress immune responses, assisting B cells in antibody production. Notably, Th1 IFN-γ-secreting cells, predominantly present in atherosclerotic lesions, contribute to plaque growth and instability. In contrast, Th2 cells that express IL-5 and IL-13 are considered to have a protective effect against atherosclerosis. Treg cells produce anti-inflammatory cytokines such as IL-10, promote macrophage exocytosis, and show a negative correlation with the development of atherosclerosis [6]. Monocytes, typically identified as CD11b^+^ CD115^+^ cells, are primarily found in bone marrow, blood, and various body tissues [7,8]. Based on differences in surface marker expression and functional characteristics, monocytes can be categorized as “classical” (Ly6C^high^) and “non-classical” (Ly6C^low^) monocytes [9]. “Classical” (Ly6C^high^) monocytes are often characterized as pro-inflammatory due to their role in generating inflammatory M1 macrophages and dendritic cells in various infection and non-infection scenarios. Conversely, “non-classical” monocytes (Ly6C^low^) are considered anti-inflammatory and have demonstrated atheroprotective effects in mouse models of atherosclerosis [10]. Various studies support the idea that Ly6C^high^ monocytes can be converted into Ly6C^low^ monocytes in the blood [11]. However, it remains unclear whether Ly6C^low^ monocytes play a role in clearing lipids, dead or dying cells, and immune complexes from the vascular endothelium [12]. 

The current therapeutic approach for atherosclerosis primarily encompasses lifestyle modifications and pharmacological interventions. Existing research indicates that weight reduction, dietary adjustments, increased physical activity, and the limitation of smoking and alcohol intake can significantly ameliorate dyslipidemia and mitigate the risk of atherosclerotic cardiovascular disease (CVD) [13,14]. In the realm of pharmacotherapy, statins remain the first-line agents for managing lipid metabolism disorders [15]; however, they are not without limitations. These include suboptimal patient adherence, intolerance manifested as muscle pain and other adverse effects, as well as potential drug interactions—particularly with macrolides—that may elevate plasma concentrations and toxicity [16,17,18]. Non-statin alternatives such as ezetimibe and novel proprotein convertase subtilisin/kexin type 9 (PCSK9) inhibitors [19] can partially mitigate these issues. However, lipid-lowering therapy offers only modest reductions in cardiovascular risk, with many patients still experiencing cardiovascular events. Surgical interventions like percutaneous coronary intervention, endarterectomy, or surgical bypass grafting carry inherent risks of stent restenosis or lumen thrombosis [20,21]. Despite mounting evidence suggesting that vascular inflammatory responses may contribute to the residual risk of atherosclerotic disease, there is currently a lack of clinical therapies directly targeting the inflammatory response within atherosclerotic lesions [22]. 

Peptide vaccines have recently emerged as a compelling and promising strategy for addressing atherosclerosis, offering advantages such as antigen specificity, preservation of the host’s immune defenses, and provision of long-term protection. Current atherosclerosis vaccines, whether aimed at stimulating antibody production or regulatory T cell responses, primarily target LDL-related factors in order to reduce plasma/serum cholesterol levels [23,24,25]. For example, vaccine-induced antibodies targeting PCSK9 (AT04A) have demonstrated efficacy in attenuating atherosclerosis in animal models [26] and exhibited significant LDL-lowering effects in a phase I clinical trial (NCT 02508896) [27]. Additionally, a series of peptides derived from the autoantigen ApoB 100, such as p18, p210, and p45, have been found to activate Treg cells and alleviate atherosclerosis in *Apoe^−/−^* or human ApoB 100 transgenic mice [28,29,30,31]. Numerous studies have also demonstrated the crucial protective role of arteriosclerosis-related immunity, yet the specific antigen responsible for eliciting this immune response remains unidentified. For instance, immune responses targeting low-density lipoprotein components, among other potential disease-related immune responses, may collaborate with a multitude of other immune responses to activate endogenous or microbial antigens that confer protection against arteriosclerosis in Treg populations [25]. These findings emphasize the necessity to explore innovative treatment strategies for atherosclerotic disease.

In a previous study, we utilized phage display library techniques and bioinformatics analysis to identify COL6A6 as a novel protective antigen associated with atherosclerosis from the serum of patients with the condition [32]. However, the specific immune mechanism by which the COL6A6 peptide vaccine provides protection against atherosclerosis remains unclear. In this study, we redesigned the COL6A6 peptide vaccine as the Pep_A6 vaccine. This vaccine consists of the COL6A6 peptide-KLH (keyhole limpet hemocyanin) conjugate and aluminum hydroxide adjuvant. Our aim was to assess the effectiveness of the Pep_A6 vaccine with emphasis on immune responses and lipid metabolism.

## 2. Materials and Methods

### 2.1. Peptide Preparation

The COL6A6 peptide (DSGPEYADVVFLVDSSDHLGLKS) was synthesized by ChinaPeptides (Suzhou, China) and conjugated with KLH at a ratio of 1:3 (*w*:*w*). The homology of this peptide with mice reached up to 91.3%.

### 2.2. Animal Vaccination

Six-week-old male *Apoe^−/−^* mice (purchased from the GemPharmatech, Nanjing, China) were utilized for vaccination. The mice were intraperitoneally injected with the Pep_A6 vaccine (60 µg/120 µL per injection) at 6 and 9 weeks of age, following the protocol established by Gunilla Nordin Fredrikson et al. [33]. Equal amounts of KLH protein and alum mixture (KLH + Alum) or phosphate buffer (PBS) were intraperitoneally administered at 6 and 9 weeks of age as negative controls for the vaccine. Two weeks after the second booster immunization, all groups were subjected to a high-fat diet (approximately 4700 kcal/kg, 17% of calories from protein, 43% from carbohydrates, and 41% from fat) for a duration of 20 weeks. The Chow group of *Apoe^−/−^* mice was continuously fed a normal diet throughout the study period. All mice were sacrificed at 30 weeks for subsequent analyses.

The animals were provided with ad libitum access to water and food, and were housed in conditions with a 12 h light/12 h dark cycle. The study design was approved by the Ethics Board of Southern Medical University.

### 2.3. Staining of the Aorta

Upon termination, all mice were humanely euthanized by exsanguination. The aortas were then perfused with PBS followed by 4% paraformaldehyde solution (a general-purpose tissue fixative, Biosharp, Hefei, China). Subsequently, the aortic arch was carefully isolated from surrounding connective tissues and mounted on a slide for Oil Red O staining (Sigma, St. Louis, Germany). A quantitative analysis of atherosclerotic plaques was conducted using a computer-aided microscopy system equipped with the Image Pro Plus 6.0 software package (Zeiss, Oberkochen, Germany) [34]. 

### 2.4. Detection of Serum IgG and IgG1

The serum samples were collected and left at room temperature for 4 h, followed by centrifugation at 3000 rpm for 15 min to separate the serum from blood cells. The total IgG level was quantified using an ELISA detection kit (Elabscience, Wuhan, China) according to the manufacturer’s instructions. The levels of Pep_A6-specific antibodies were determined by coating 96-well ELISA plates (Costar, Corning, NY, USA) with Pep_A6 (2 µg/mL) diluted in a NaHCO_3_/Na_2_CO_3_ buffer (pH 9.0) (Solarbio, Beijing, China). Bovine serum albumin (BSA, Genview, Beijing, China) at a concentration of 2 µg/mL was coated as a negative control. To further characterize the IgG subtypes of antibodies against Pep_A6, HRP-conjugated goat anti-mouse IgG (Multisciences, GAM007) and IgG1 (Invitrogen, A10551) detection antibodies were employed sequentially, followed by TMB substrate and stop solutions. The optical density at 450 nm (OD 450) was measured using a microplate reader (Varioskan Flash, Thermo Fisher, Waltham, MA, USA). The standard curve was generated using the ELISACalc software V1.0 package (Boster, Wuhan, China), and concentration conversions were performed.

### 2.5. Cell Isolation, Cell Staining, and Flow Cytometry

Mouse spleens were aseptically processed to obtain single-cell suspensions in the designated cell culture medium. For Th cell polarization assessment, a portion of spleen cells was seeded at a density of 1–2 × 10^6^ cells/mL/dish and stimulated with 2 µL of Cell Activation Cocktail (with Brefeldin A) (BioLegend, San Diego, CA, USA) per milliliter of cell suspension for 6 h at 37 °C. The cells were then collected and stained with CD4-FITC, IFN-γ-BV421, and IL-4-PE/Cy7 according to the manufacturer’s instructions. Treg cells were detected by staining another portion of spleen cells with CD4-FITC, CD25-APC, and FOXP3-PE. Peripheral blood mononuclear cells were stained with CD11b-PE, CD115-APC, and Ly6C-Brilliant Violet 421™ to evaluate the Ly6C^high^ and Ly6C^low^ monocyte populations. Following two washes with cell staining buffer, all cell samples underwent a flow cytometry analysis (BD Fortessa II, FACSVerse or Canto II), with data analyzed using the FlowJo V10 software package (Stanford, Palo Alto, CA, USA). Details of all antibodies are provided in Table S2.

### 2.6. Four Blood Lipid Tests

Serum samples were collected and subsequently centrifuged at 3000 rpm for 15 min at room temperature to separate serum from blood cells. The levels of serum TG, CHO, HDL-C, and LDL-C were quantified using the Jiancheng Bioengineering Institute kit according to the manufacturer’s instructions. 

### 2.7. Histological Analysis

The murine hearts were embedded in OCT compound (Sakura, Tokyo, Japan), and cryosections (10 µm, Thermo, Waltham, MA, USA) of the aortic root containing the three aortic valves were prepared. Cryosections were stained with Oil Red O (Biossci, Wuhan, China) to evaluate plaque size and Masson’s trichrome (Biossci, Wuhan, China) to assess collagen and fibroblast content. Similarly, the liver was embedded in OCT compound, and cryosections were stained with Oil Red O. Histological quantification was conducted using an Olympus upright microscope and a Hamamatsu Imaging System (Olympus, Tokyo, Japan), with data analyzed using NDP.view 2.9.22 RUO software (Hamamatsu Photonics, Hamamatsu, Japan).

### 2.8. RNA-Seq of Mouse Liver

Total RNA (1 µg) from mouse liver tissues was utilized for the preparation of sequencing libraries using the NEB-Next^®^ UltraTM RNA Library Prep Kit for Illumina^®^ (NEB, Belverley, MA, USA). RNA integrity was assessed using the RNA Nano 6000 Assay Kit of the Bioanalyzer 2100 system (Agilent Technologies, Palo Alto, CA, USA). Index-coded samples were clustered on a cBot Cluster Generation System with TruSeq PE Cluster Kit v3-cBot-HS prior to sequencing on an Illumina Novaseq platform. DESeq2 (1.16.1) in R package was employed for conducting differential expression analysis of two conditions/groups, each with two biological replicates per condition. Genes exhibiting an adjusted *p*-value < 0.05 were identified as differentially expressed. Furthermore, a Gene Ontology (GO) enrichment analysis of differentially expressed genes (DEGs) was performed using the R package clusterProfiler, and GO terms with corrected *p*-values below 0.05 were considered significantly enriched. Additionally, the R package clusterProfiler was used to assess the enrichment of DEGs in Kyoto Encyclopedia of Genes and Genomes (KEGG) pathways. The data generated from this study have been deposited in the Gene Expression Omnibus (GEO) database under reference number GSE245813.

### 2.9. RNA Isolation, cDNA Synthesis, and Quantitative Polymerase Chain Reaction (qPCR)

The RNA was isolated using RNAiso Plus (Takara, Osaka City, Japan) following the manufacturer’s instructions. Subsequently, a cDNA synthesis was carried out with HiScript^®^ III RT SuperMix for qPCR (+gDNA wiper) (Vazyme, Nanjing, China). The qPCR analysis was conducted using specific primers listed in Table S3 and ChamQ SYBR qPCR Master Mix (Low ROX Premixed) (Vazyme, Nanjing, China) on a Quant Studio5 Real-Time PCR System. Relative gene expression levels were calculated by comparison with the endogenous control gene Gapdh.

### 2.10. Statistical Analysis

GraphPad Prism 8.0 (San Diego, CA, USA) was used for data visualization, statistical analysis, and chart creation. Welch’s correction for unequal variances, one-way analysis of variance (ANOVA) followed by Tukey’s multiple comparison test, or Games Howell’s multiple comparison test were applied as appropriate. Data were presented as mean ± SEM and statistical significance was defined as a *p*-value less than 0.05.

## 3. Results

### 3.1. Pep_A6 Vaccination Significantly Reduces Plaque Area in Apoe^−/−^ Mice

**Figure 1:**
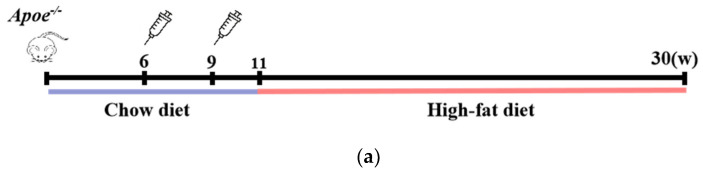

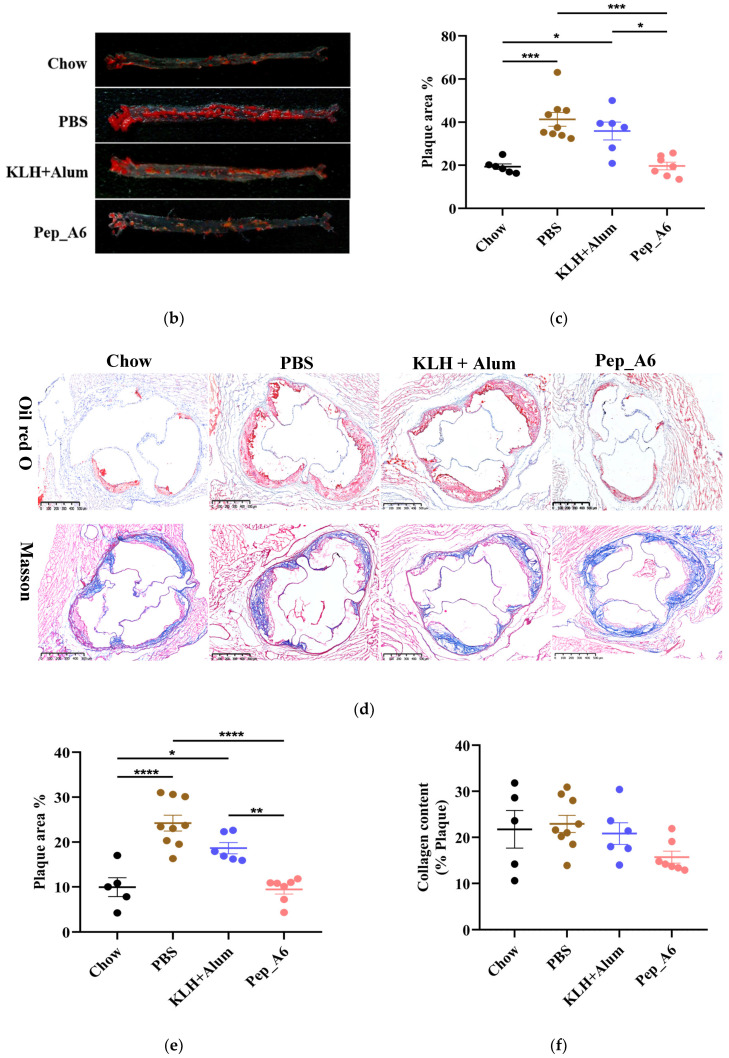
Pep_A6 vaccination alleviated atherosclerotic lesions in hyperlipidemic *Apoe^−/−^* mice. (**a**) Experimental scheme for high-fat diet-induced mouse model and vaccine immunization strategy. The Chow group was fed an normal diet throughout the whole course. (**b**) Aortic Oil Red O staining of different groups. (**c**) Percentage of plaque area of the entire artery. (**d**) Oil red O staining and Masson staining of the aortic valve (5×, scale bar = 500 µm). (**e**,**f**) Quantification of plaque staining area at the aortic valve. (Chow: *n* = 5−6; PBS: *n* = 9; KLH + Alum: *n* = 6; Pep_A6: *n* = 7. Data are expressed as mean ± SEM. * *p* < 0.05, ** *p* < 0.01, *** *p* < 0.001, **** *p* < 0.0001).

Male *Apoe^−/−^* mice, aged 6 weeks, were intraperitoneally administered the Pep_A6 vaccine, (KLH + Alum) mixture, or PBS to evaluate plaque progression after a 20-week high-fat diet. The Chow group received a normal diet throughout the study. Figure 1a illustrates the experimental scheme. The plaque area in mice injected with PBS increased significantly by 53% compared to the Chow group. In contrast, the Pep_A6 vaccine group exhibited a significant 50% reduction in plaque area compared to the PBS group and the (KLH + Alum) group. There was no significant difference in plaque area between the (KLH + Alum) and PBS groups (Figure 1b,c). Figure 1d presents Oil Red O and Masson trichrome staining of the aortic root in each group. The Oil Red O staining of the aortic root revealed a significant increase in plaque area in the PBS group compared to the Chow group, while the Pep_A6 vaccine group exhibited a significant reduction in plaque area induced by the high-fat diet (Figure 1e). Masson trichrome staining showed no significant differences in collagen content within aortic root plaques across all groups, suggesting that the Pep_A6 vaccine did not impact plaque stability (Figure 1f).

### 3.2. Pep_A6 Vaccination Affects the Serum Level of IgG Antibodies and Produces Specific Antibodies

**Figure 2:**
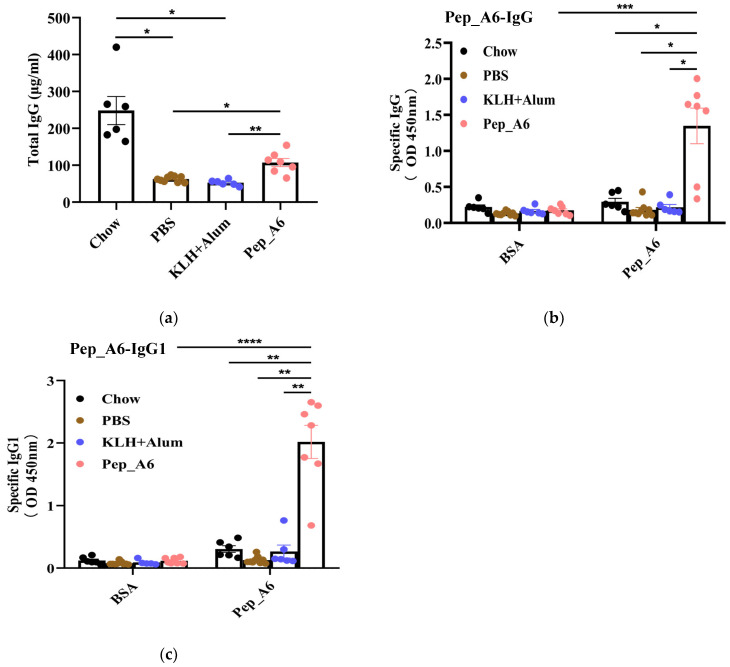
Pep_A6 vaccination produced antibodies specifically targeting Pep_A6. HFD mice were intraperitoneally treated with PBS, KLH + Alum mixture, or Pep_A6 vaccine. (**a**) Serum levels of total IgG were quantified using capture ELISA in different treatment groups. Levels of Pep_A6-specific (**b**) IgG and (**c**) IgG1 in serum from different treatment groups are shown. (Chow: *n* = 6; PBS: *n* = 9; KLH + Alum: *n* = 6; Pep_A6: *n* = 6−7. Data are expressed as mean ± SEM. * *p* < 0.05, ** *p* < 0.01, *** *p* < 0.001, **** *p* < 0.0001).

After completion of the study, the total serum IgG levels were evaluated. Remarkably, the Chow group, which was fed a normal diet, demonstrated significantly elevated levels of IgG antibody compared to the other groups. As illustrated in Figure 2a, the serum IgG levels in the Pep_A6 vaccine group showed a significant increase compared to HFD mice injected with PBS or the (KLH + Alum) mixture. To assess whether antibodies produced by Pep_A6 vaccination specifically recognized Pep_A6, Pep_A6 was immobilized on a 96-well plate. In comparison to all other groups, serum levels of Pep_A6-specific IgG and IgG1 were significantly elevated (Figure 2b,c), suggesting that the immune response induced by the vaccine is skewed towards a Th2 cell profile.

### 3.3. Pep_A6 Vaccination Suppresses Th1 Cell Differentiation but Increases Treg Cell Proportion

**Figure 3:**
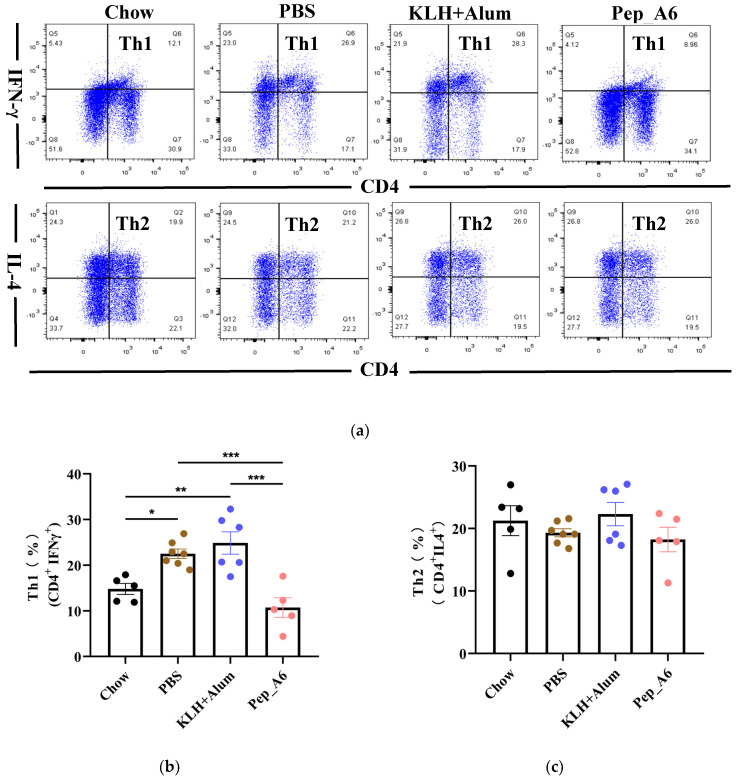

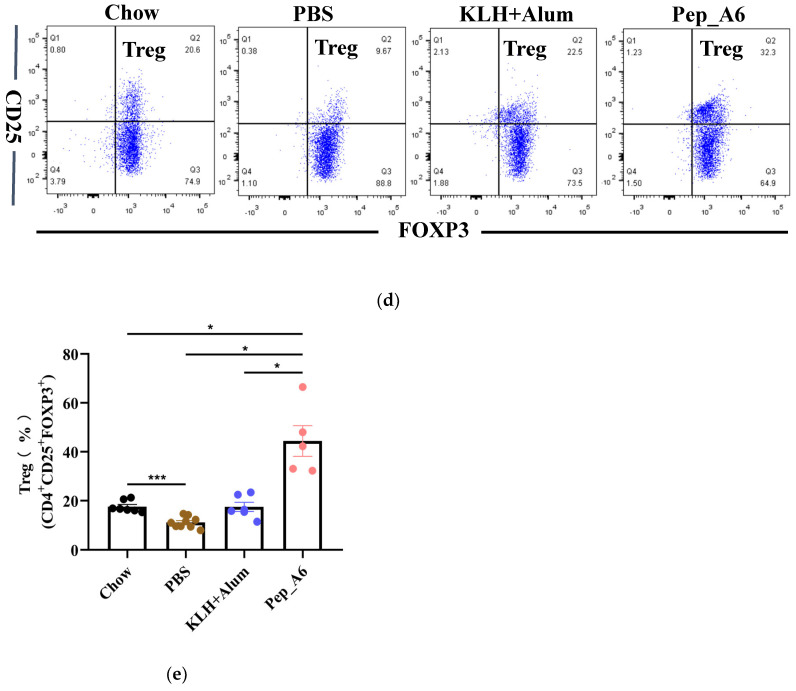
Pep_A6 vaccination suppressed Th1 cell differentiation but increased Treg cell proportion. (**a**) Flow cytometry analysis of splenic Th1 and Th2 cells in different treatment groups. (**b**) Percentage of Th1 (CD4^+^IFN-γ^+^) cells in different groups. (**c**) Percentage of Th2 (CD4^+^IL-4^+^) cells in different groups. (**d**) Flow cytometry analysis of splenic Treg cells. (**e**) Percentage of Treg (CD4^+^CD25^+^FOXP3^+^) cells in different groups. (Chow: *n* = 5−7; PBS: *n* = 7−9; KLH + Alum: *n* = 6; Pep_A6: *n* = 5. Data are expressed as mean ± SEM. * *p* < 0.05, ** *p* < 0.01, *** *p* < 0.001).

Th cells play a crucial role in assessing the immune response to the vaccine. We evaluated the differentiation of Th cells isolated from the spleens of *Apoe^−/−^* mice, using CD4, IFN-γ, and IL-4 as markers for Th1 and Th2 cells (Figure 3a). Flow cytometry analysis of single-cell suspensions from the mice spleens revealed a significant decrease in Th1 cells (CD4^+^IFN-γ^+^) in the Pep_A6 vaccine group compared to both the PBS group and the (KLH + Alum) group (Figure 3b). However, there was no significant difference in Th2 cell population among these groups (Figure 3c). Additionally, we employed CD4, CD25, and FOXP3 as markers to determine the proportion of Treg cells derived from spleens (Figure 3d). Our findings indicated a notable increase in Treg cells in the Pep_A6 vaccine group compared to the PBS group (Figure 3e). 

### 3.4. Pep_A6 Vaccination Promotes Differentiation of Anti-Inflammatory Ly6C^low^ Monocytes

**Figure 4:**
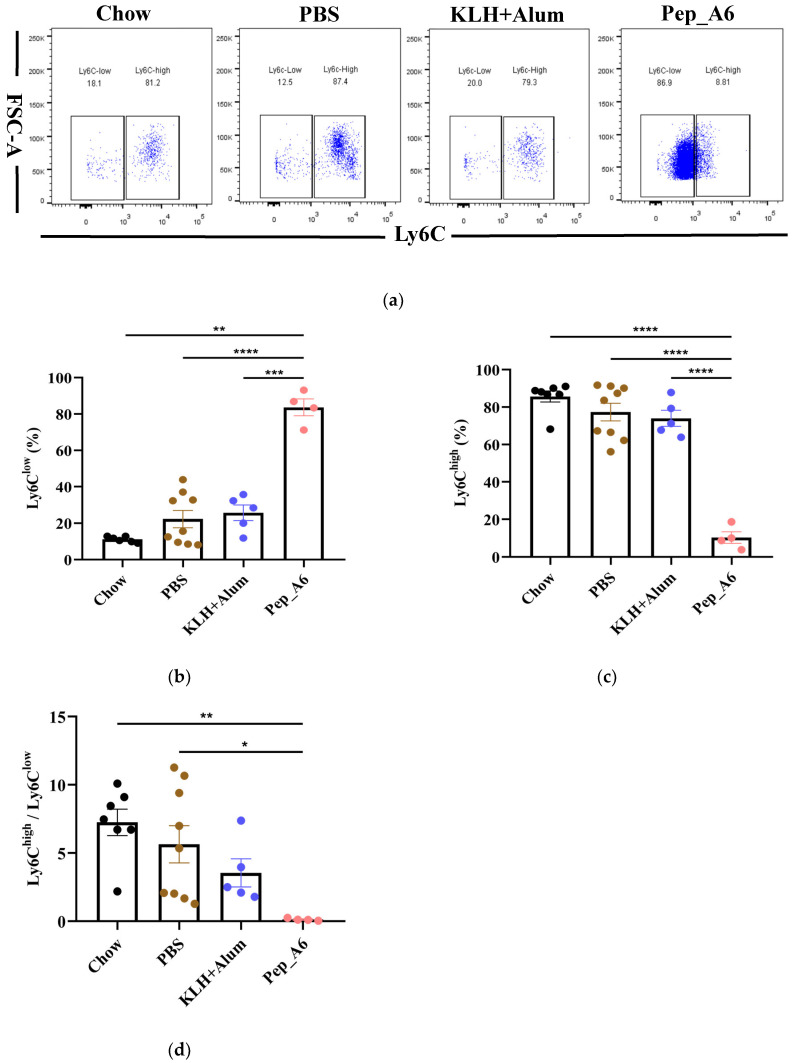
Pep_A6 vaccination significantly increased anti-inflammatory Ly6C^low^ monocytes. (**a**) Flow cytometry analyzed peripheral blood monocytes in different groups. Percentage of (**b**) Ly6C^low^ (CD115^+^CD11b^+^Ly6C^−^) and (**c**) Ly6C^high^ (CD115^+^CD11b^+^Ly6C^+^) monocytes in different groups. (**d**) The ratio of Ly-6C^high^/Ly-6C^low^ monocytes in the blood. (Chow: *n* = 7; PBS: *n* = 9; KLH + Alum: *n* = 5−6; Pep_A6: *n* = 4−5. Data are expressed as mean ± SEM. * *p* < 0.05, ** *p* < 0.01, *** *p* < 0.001, **** *p* < 0.0001).

The progression of atherosclerotic plaque is closely associated with the pro-inflammatory or anti-inflammatory effects of monocytes; thus, we investigated the impact of the Pep_A6 vaccine on monocyte differentiation in mice. The murine-circulating monocytes comprise two major subsets: Ly6C^high^(pro-inflammatory) and Ly6C^low^(anti-inflammatory) cells. Our findings revealed that the Pep_A6 vaccine significantly promoted the differentiation of Ly6C^low^ monocytes (CD11b^+^CD115^+^Ly6C^−^) while reducing the abundance of Ly6C^high^ monocytes (CD11b^+^CD115^+^Ly6C^+^) in the bloodstream (Figure 4a–c). As a result, the ratio of Ly6C^high^/Ly6C^low^ in the Pep_A6 vaccine group was decreased compared to the other three groups (Figure 4d), indicating an anti-inflammatory effect of the vaccine. 

### 3.5. Pep_A6 Vaccination Significantly Decreases Serum LDL-C Concentrations and Hepatic Lipid Accumulation in Apoe^−/−^ Mice

**Figure 5:**
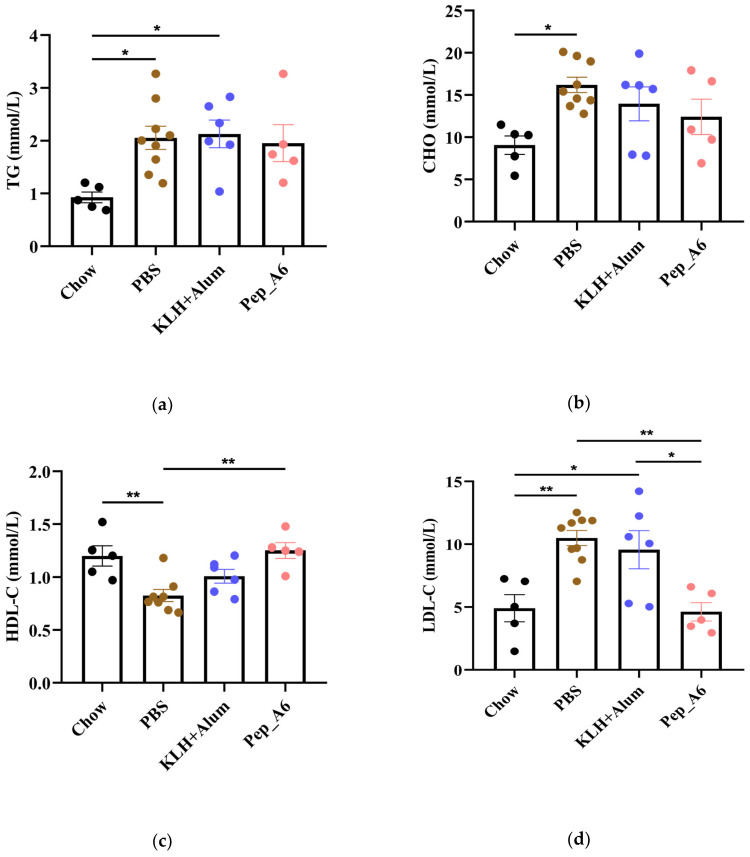

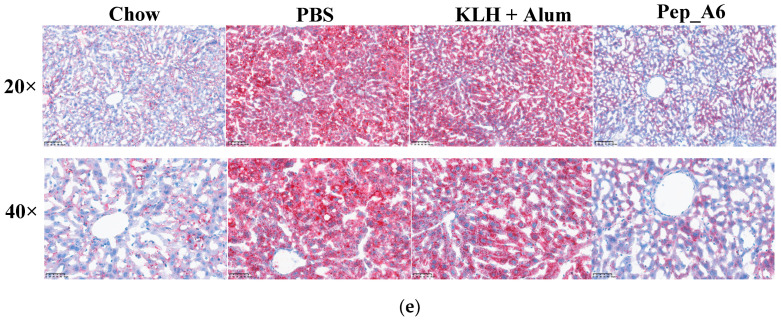
Pep_A6 Vaccination significantly decreased serum LDL-C concentrations and hepatic lipid accumulation. (**a**–**d**) Serum TG levels, serum CHO levels, serum HDL-C levels, and serum LDL-C levels were measured in different groups. (Chow: *n* = 5; PBS: *n* = 9; KLH + Alum: *n* = 6; Pep_A6: *n* = 5. Data are expressed as mean ± SEM. * *p* < 0.05, ** *p* < 0.01.) (**e**) Histopathological observation of Oil Red O staining in the liver of mice (20×, scale bar = 100 µm; 40×, scale bar = 50 µm).

In Figure 5a,b, the HFD mice in the PBS group exhibited elevated serum TG and CHO levels compared to the Chow mice. The Pep_A6 vaccine did not show a significant effect on the expression of serum TG and CHO. HFD induction led to a reduction in HDL-C levels in the PBS group, which was restored by the Pep_A6 vaccine (Figure 5c). Importantly, serum LDL-C levels were markedly reduced in mice immunized with the Pep_A6 vaccine compared to HFD mice injected with PBS or the (KLH + Alum) mixture (Figure 5d). Lipid metabolism is associated with liver function. Therefore, we conducted a histopathological analysis of mouse livers in each group and stained them with Oil Red O to observe the status of liver lipids status. Oil Red O staining revealed that lipid droplet accumulation in the livers of HFD mice injected with PBS or the (KLH + Alum) mixture was significantly increased compared to Chow mice. In contrast, the livers of mice immunized with the Pep_A6 vaccine exhibited significantly less lipid accumulation than those of HFD mice (Figure 5e).

### 3.6. Pep_A6 Vaccination Partly Restores the Expression of Lipid Metabolism-Related Genes Influenced by a High-Fat Diet

**Figure 6:**
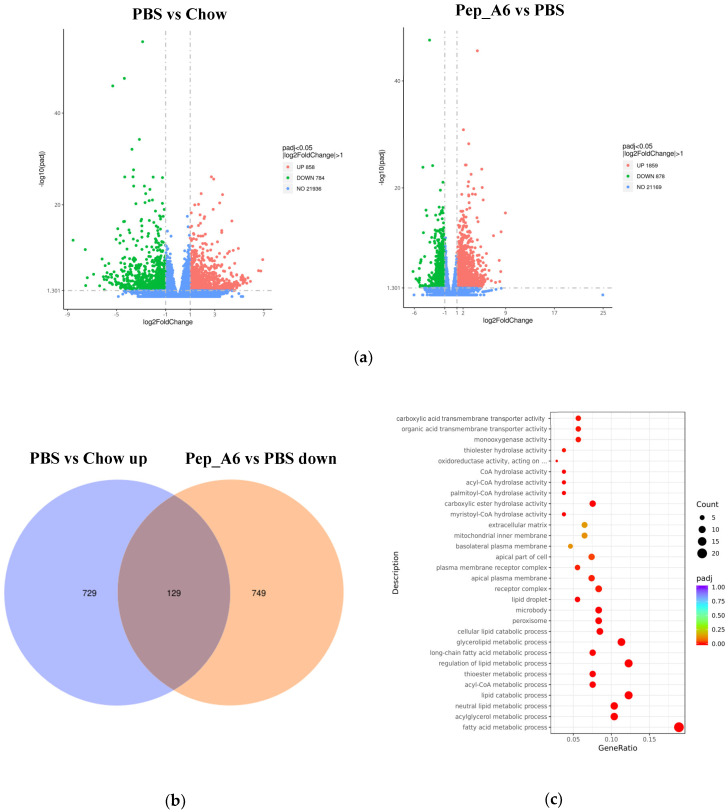

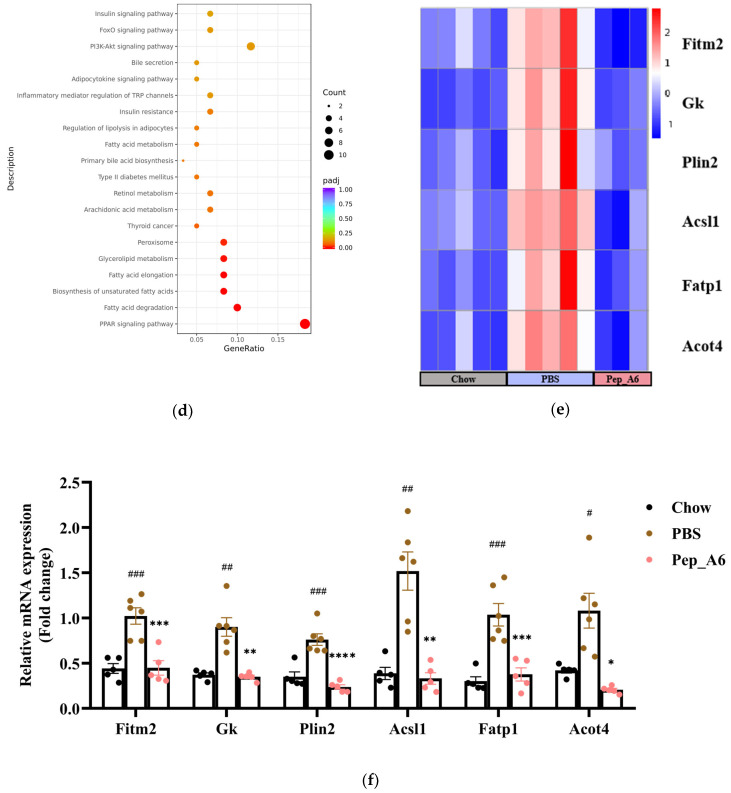
Pep_A6 Vaccination partially restored the expression of liver lipid metabolism-related genes up-regulated by a high-fat diet. (**a**) Volcano plots depicting differentially expressed genes (DEGs) in the PBS group compared to the Chow group, and in the Pep_A6 vaccine group compared to the PBS group (*n* = 3−5, padj < 0.05). The red dots indicate gene up-regulation, and the green dots indicate gene down-regulation. (**b**) Venn plot showing overlapping genes between up-regulated from the HFD mice injected with PBS relative to the Chow mice and down-regulated from the Pep_A6-immunized mice relative to the HFD mice injected with PBS. Blue represents the up-regulated differential genes in the PBS group compared with Chow group, orange represents the down-regulated differential genes in the vaccine group compared with PBS group, and the region in the middle represents the overlapping DEGs between the two comparison combinations. (**c**) Bubble plot of GO enrichment analysis of overlapping DEGs down-regulated in vaccine groups (padj < 0.05); the bubble color from red to purple in the plot indicates the significance degree of gene enrichment. (**d**) Bubble plot of KEGG enrichment analysis of overlapping DEGs (padj < 0.05); the bubble color from red to purple in the plot indicates the significance degree of gene enrichment. (**e**) Heatmaps displaying top-ranked genes related to lipid metabolism among the 129 overlapping DEGs. Red indicates gene upregulation, while blue indicates gene downregulation. (**f**) Quantitative polymerase chain reaction (qPCR) expression analysis of *Fitm2*, *Gk*, *Plin2*, *Acsl1*, *Fatp1*, and *Acot4*. (Chow: *n* = 5; PBS: *n* = 6; Pep_A6: *n* = 5. Data are expressed as mean ± SEM. ^#^ *p* < 0.05, ^##^ *p* < 0.01, ^###^ *p* < 0.001, as compared with the Chow group; * *p* < 0.05, ** *p* < 0.01, *** *p* < 0.001, **** *p* < 0.0001, as compared with the PBS group).

To investigate the molecular mechanisms underlying the effects of the Pep_A6 vaccine on the liver, RNA sequencing was conducted with 3–5 replicates per group. Volcano plots revealed the significant up-regulation of 858 genes and down-regulation of 784 genes in HFD mice injected with PBS compared to Chow mice (Figure 6a left). In comparison, immunization with the Pep_A6 vaccine resulted in the up-regulation of 1859 genes and down-regulation of 878 genes compared to HFD mice injected with PBS (Figure 6a right). The Venn plot identified 129 overlapping DEGs between those up-regulated in HFD mice injected with PBS relative to Chow mice and those down-regulated in mice immunized with the Pep_A6 vaccine relative to HFD mice injected with PBS (Figure 6b). The results suggest that the Pep_A6 vaccine can suppress the upregulation of 129 genes in the liver of mice following a high-fat diet. To achieve a more comprehensive understanding of the 129 overlapping genes, we performed a GO functional enrichment analysis. The GO database provides a comprehensive collection of gene functions, encompassing three primary categories: biological processes, cellular components, and molecular functions. Based on the results of functional enrichment analysis, we identified the 30 most significant terms for generating bubble plots, as illustrated in Figure 6c. Notably, among the 129 overlapping genes downregulated by the Pep_A6 vaccine, there was a particularly pronounced enrichment of the fatty acid metabolism process. Subsequently, we performed a KEGG pathway enrichment analysis. The KEGG is a comprehensive database that integrates genetic, chemical, and system functional information. The significance threshold for KEGG functional enrichment is padj < 0.05. We identified the top 20 most significant KEGG pathways from the results of functional enrichment to generate a bubble plot in Figure 6d, which clearly demonstrated PPAR signaling pathway as the most enriched pathway. Based on the GO and KEGG analysis results, we identified the top-ranked DEGs associated with LDL-C metabolism, atherosclerosis, or liver lipid deposition and constructed a heatmap as shown in Figure 6e. The heatmap visualization showed the up-regulation of specific genes (*Fitm2*, *Gk*, *Plin2*, *Acsl1*, *Fatp1*, *Acot4*) in HFD mice injected with PBS and their down-regulation in mice immunized with the Pep_A6 vaccine. Furthermore, the results of the qPCR verification (Figure 6f) were basically consistent with the transcriptome results. In comparison to the Chow group fed a normal diet, the expression levels of various genes in the PBS group fed a high-fat diet were significantly upregulated in the liver of mice, while the vaccine group showed significant downregulation compared to the PBS group.

## 4. Discussion

It has been confirmed type VI collagen is predominantly located on the fibrous cap, and its content increases with plaque progression [35]. COL VI is composed of six peptide chains, designated as α1, α2, α3, α4, α5, and α6, which are encoded by COL6A1~COL6A6 genes. The N-terminal of the α6 peptide chain primarily consists of seven von Willebrand factor A (VWA) domains, while the C-terminal contains two or three VWA domains and one or two unique sequences. The collagen triple helix domain is situated between the N- and C-terminal regions [36]. In this study, a synthesized COL6A6 peptide was obtained from the peripheral blood of atherosclerosis patients. The sequence corresponds to the N-terminal of the α6 peptide chain and comprises 23 amino acids. Notably, this polypeptide sequence showed high homology (91.3%) in both humans and mice with only two amino acid variations (Table S1), which avoided issues related to autoimmune rejection when immunizing animal models. The immunization protocol adopted was consistent with the successful example of ApoB-100 [31,34]. Primary immunization was administered at six weeks of age followed by a booster dose three weeks later, each mouse receiving at least 50 µg of vaccine per dose.

It has been established that antigen-specific immune protection can be achieved through various mechanisms, including the production of protective antibodies, the suppression or inactivation of pathogenic T cell clones (inducing non-responsiveness), or the induction of suppressive cellular immunity mediated by Treg cells [29,37,38]. In recent years, two distinct types of atherosclerosis vaccines, antibody-induced and regulatory T cell-induced, have been developed and validated in animal models [39]. Both strategies primarily target LDL-related factors to reduce plasma/serum LDL levels. In this study, Pep_A6 was identified in the peripheral blood of atherosclerosis patients [32]. *Apoe^−/−^* mice were immunized with the reconstituted Pep_A6 vaccine and fed a high-fat diet for 20 weeks. The results demonstrated that this vaccination reduced atherosclerotic plaques by about 50%. On the one hand, Pep_A6 vaccination significantly increased the proportion of Treg in the spleen. On the other hand, the Pep_A6-specific antibody IgG and IgG1 levels were significantly elevated in the vaccine group. It has been reported that in mice, Th1 cells mainly mediate cellular immune response, while Th2 cells mainly participate in humoral immunity and promote the secretion of IgG1 [28,40]. In this study, we observed a significant increase in the Pep_A6-specific IgG1 in the vaccine group, indicating a bias towards Th2 cell-mediated humoral immunity. Additionally, our findings showed that Pep_A6 vaccination led to a significant reduction in serum LDL levels and an increase in HDL-C levels. These results suggest that the Pep_A6 vaccine may elicit a combined response involving both antibody-induced and regulatory T cell-induced mechanisms which effectively modulate blood lipid levels.

The efficacy and safety of the vaccine depend on the proper activation of T cells and B cells by the target and carrier protein peptide sequences. The carrier protein in the Pep_A6 vaccine is KLH, known for its high immunogenicity, promoting CD4^+^ T cell differentiation and facilitating antibody production [41]. Adjuvants play a role in determining CD4^+^ Th cell differentiation during vaccination [42]. For example, CpG adjuvants tend to induce Th1 differentiation, leading to antibody-mediated cytotoxic responses involving CD8^+^ T cells. Conversely, the alum adjuvant used in the Pep_A6 vaccine primarily activates Th2 differentiation and mitigates cytotoxic damage. Previous animal experiments have demonstrated that Th1 cells have a pro-atherogenic effect, including studies with IFN-γ deficient mice [43,44,45] and mice genetically deficient in the Th1 transcription factor T-bet [46], all of which exhibited reduced atherosclerosis development. It has been reported that Treg cells can prevent T cells from differentiating into Th1 subtypes, thereby limiting their pathogenic activity [47]. These findings suggest that the Pep_A6 vaccination probably inhibits Th1 cell differentiation by increasing the vaccine-induced Treg cells, thus reducing vascular inflammation and ameliorating atherosclerosis. Nevertheless, we did not transfer Treg cells as conducted by Hafid Ait Oufell et al. [48] and Adi Mor et al. [49] to ascertain the specific impact of Treg cells. This aspect will be addressed in future research.

The synthesis of fatty acids and fats in the liver is a highly regulated metabolic pathway, and genes related to lipid metabolism enable coordinated regulation at the transcriptional level [50]. In this study, the qPCR results revealed that in the high-fat diet environment, there was significant up-regulation of mRNA expressions of *Fitm2, Gk, Plin2, Acsl1, Fatp1*, and *Acot4* genes identified as significantly enriched in transcriptome sequencing in the PBS group. Following immunization with the Pep_A6 vaccine, the expression levels of these genes was significantly down-regulated to match those observed in the Chow group with normal diet. In particular, the mRNA expression of *Acsl1* in the Pep_A6 vaccine group was notably lower than that observed in Chow group. Furthermore, a marked reduction in hepatic lipid accumulation was observed through liver oil red O staining in the vaccine group. It is evident that the Pep_A6 vaccine demonstrates a certain ability to regulate lipid metabolism and relies on key genes. According to numerous research reports, these genes are known to play a pivotal regulatory role in the pathogenesis of diseases such as hepatic steatosis, atherosclerosis, and hyperlipidemia. Prior studies have shown that macrophage-specific expression of *Acsl1* plays a pivotal role in exacerbating atherosclerosis in diabetic mice, and the adoptive transfer of *Acsl1^−/−^* macrophages may represent a potential therapeutic strategy for atherosclerosis [51]. Following *Acot4* overexpression, there was a significant upregulation of free fatty acid levels in the plasma and liver of mice, leading to increased lipid accumulation in the liver and subsequent hepatic steatosis [52]. *Gk*, or glycerol kinase, has been shown to enhance liver CHO and TG expression, as well as plasma LDL levels [53]. *Fatp1* is closely related to intracellular lipid deposition. For example, knocking down *Fatp1* in 3T3-L1 fat cells reduced TG accumulation and droplet size [54]. Previous studies have reported that *Plin2^−/−^* mice exhibited a reduction of more than 60% in serum TG content and were protected from fatty liver disease [55]. *Fitm2* is widely recognized as a fat storage-induced transmembrane protein 2. The overexpression of *Fitm2* genes resulted in a marked increase in neutral lipid content in fat cells [56]. These studies have confirmed that the overexpression of *Fitm2, Gk, Plin2, Acsl1, Fatp1*, and *Acot4* genes exacerbates cellular lipid accumulation, elevates serum LDL levels, and accelerates the progression of atherosclerosis. Conversely, the knockout or downregulation of these genes effectively alleviates the corresponding symptoms. This finding is consistent with our study’s observation that the Pep_A6 vaccine can effectively downregulate the expression of these genes in a high-fat environment, suggesting that the Pep_A6 vaccine may play a positive regulatory role by restoring the expression of these key lipid metabolism-related genes. 

Upon further literature review, it was determined that the genes Acsl1, Acot4, Fatp1, and Plin2 are all under the regulation of PPARα. PPARα is a ligand-activated nuclear receptor with high expression in the liver [57] and plays a crucial role in regulating genes associated with hepatic lipid metabolism, including the activation, elongation, uptake, and triglyceride formation of fatty acids within the cell [58]. Yenna Lee et al.’s study demonstrated that PPARα agonists can mitigate the progression of atherosclerosis and the exacerbation of hepatic steatosis in *Apoe^−/−^FXR^−/−^* mice by promoting beta-oxidation, fatty acid uptake, and triglyceride hydrolysis [59]. In rodent models of systemic inflammation, atherosclerosis, and non-alcoholic steatohepatitis (NASH), PPARα exerts negative regulation on pro-inflammatory and acute phase response signaling pathways [60,61]. The above findings further suggest that the Pep_A6 vaccine may modulate PPARα to downregulate the expression of lipid metabolism-related genes, thereby effectively reducing the level of LDL-C in mouse serum, inhibiting liver lipid accumulation, and preventing atherosclerosis. Despite its limitations, this undoubtedly lays a solid foundation and serves as a starting point for us to delve deeper into the specific molecular mechanisms through which the Pep_A6 vaccine regulates liver lipid metabolism in future studies.

However, despite the concept of anti-atherosclerosis vaccines being proposed over 20 years ago, and the preclinical work providing positive proof-of-principle results, the transition to clinical trials has been sluggish. One common limitation of experimental atherosclerosis vaccine research is their focus on preventing the effects of early atherosclerosis [25], and this study follows suit. This situation may be analogous to the inclusion of relatively young individuals in primary prevention trials, which differs significantly from the scenario where late-stage plaques lead to clinical symptoms in patients. Hence, more research efforts are warranted to ascertain the translational potential of our experimental findings into enduring therapeutic modalities for advanced human plaques. Nevertheless, the Pep_A6 vaccine has demonstrated significant potential for translation as a novel protective vaccine against atherosclerosis. Anton Gistera et al. [62] found that low-density lipoprotein (LDL) reactive T cells play a role in the anti-LDL immune response, leading to three arteriosclerosis-protective mechanisms: antibody-dependent LDL clearance, liver immune metabolic effects, and reduced vascular inflammation. Similarly, in this study, the Pep_A6 vaccine also exhibited a synergistic effect on cellular and humoral immunity, primarily through the induction of Th2 immune responses and the stimulation of Pep_A6-specific IgG1 antibody production. Moreover, we have made the novel observation that the Pep_A6 vaccine induces an expansion of anti-inflammatory Ly6C^low^ monocytes, which can synergize with Th2 cells to exert a pivotal role in dampening vascular inflammation. On the other hand, our study shows that the Pep_A6 vaccine can regulate the expression of liver genes such as *Fitm2*, *Gk*, *Plin2*, *Acsl1*, *Fatp1*, and *Acot4*, thereby lowering serum LDL-C levels and hepatic lipid accumulation while improving atherosclerosis. Due to its multifaceted effects, the Pep_A6 vaccine, consisting of a COL6A6 peptide-KLH-conjugate and alum adjuvant, may serve as a promising vaccine formula in the future treatment of atherosclerosis.

## Data Availability

The datasets used and/or analyzed during the current study are available from the corresponding author on reasonable request.

## Supplementary Materials

The following supporting information can be downloaded at: https://www.mdpi.com/article/10.3390/cells13181589/s1, Table S1: The Comparison of amino acid sequences of COL6A6 in human and mouse; Table S2: Antibody information; Table S3: Primer sequence for qPCR; Figure S1: Blank controls for flow cytometry data from different cell types; Figure S2: Pep_A6 Vaccination partially restored the expression of liver lipid metabolism-related genes up-regulated by a high-fat diet.
