# Supplementary material for: COL6A6 Peptide Vaccine Alleviates Atherosclerosis through Inducing Immune Response and Regulating Lipid Metabolism in Apoe−/− Mice
Source: Cells. 2024 Sep 21;13(18):1589. doi: 10.3390/cells13181589 (PMC11429512; doi:10.3390/cells13181589)
Supplement: Supplementary file 1 [file cells-13-01589-s001.zip › cells-3125490-supplementary.pdf]

SUPPLEMENTAL MATERIALS

COL6A6 peptide vaccine alleviates atherosclerosis through inducing immune response and regulating lipid metabolism in *Apoe*$^{-/-}$ mice

Dongmei Tang [1], Yan Liu [1], Rui Duan [1], Run Lin [1], Zhonghao Li [1], Xianyan Liu [1], Jingrong Huang [1], Ming Zhao [1,*]

1 Department of Pathophysiology, Key Lab for Shock and Microcirculation Research of Guangdong, School of Basic Medical Sciences, Southern Medical University, Guangzhou 510515, China
* Correspondence: mzhao66@smu.edu.cn (Ming Zhao)

Table S1. The comparison of amino acid sequences of COL6A6 in human and mouse

Species	Amino acid sequence
Homo sapiens	DSGPEYADVVFLVDSSDRLGSKS
Mus musculus	DSGPEYADVVFLVDSSDHLGLKS

Table S2. Antibody information

Reagent name	brand	Cargo number
FITC anti-mouse CD4	BioLegend	100406
Brilliant Violet 421™ anti-mouse IFN-γ	BioLegend	505829
PE/Cyanine7 anti-mouse IL-4	BioLegend	504118
APC Rat Anti-Mouse CD25	BD Biosciences	557192
PE anti-mouse FOXP3	BioLegend	126404
PE anti-mouse CD11b	eBioscience	101208
APC anti-mouse CD115	BioLegend	135510
Brilliant Violet 421™ anti-mouse Ly6C	BioLegend	128031
Cell Activation Cocktail (with Brefeldin A)	BioLegend	423303

Table S3. Primer sequence for qPCR

Gene name	Forward primer	Reverse primer

Gk	ACCCTCCATGCCTGAAACAA	ACCACTTTCTGGAGACTGAGTT
Plin2	TCCACTGTCCACCTGATTGA	TGGCATGTAGTCTGGAGCTG
Acsl1	CCATCTTCCCTGTGGTTCCC	ACCCAGGCTCGACTGTATCT
Fatp1	TCACTGGCGCTGCTTTGGTT	TAGCCGAACACGAATCAGAA
Acot4	GCCTGTAACAGACATGGTAGATTC	CTGTAACAAGCACAGGCTGGTA
Fitm2	TCATTGCCCTTACCAACTACCA	AGTGGCCCGAGATGTCAAA
Gapdh	AGGTCGGTGTGAACGGATTTG	TGTAGACCATGTAGTTGAGGTCA

A

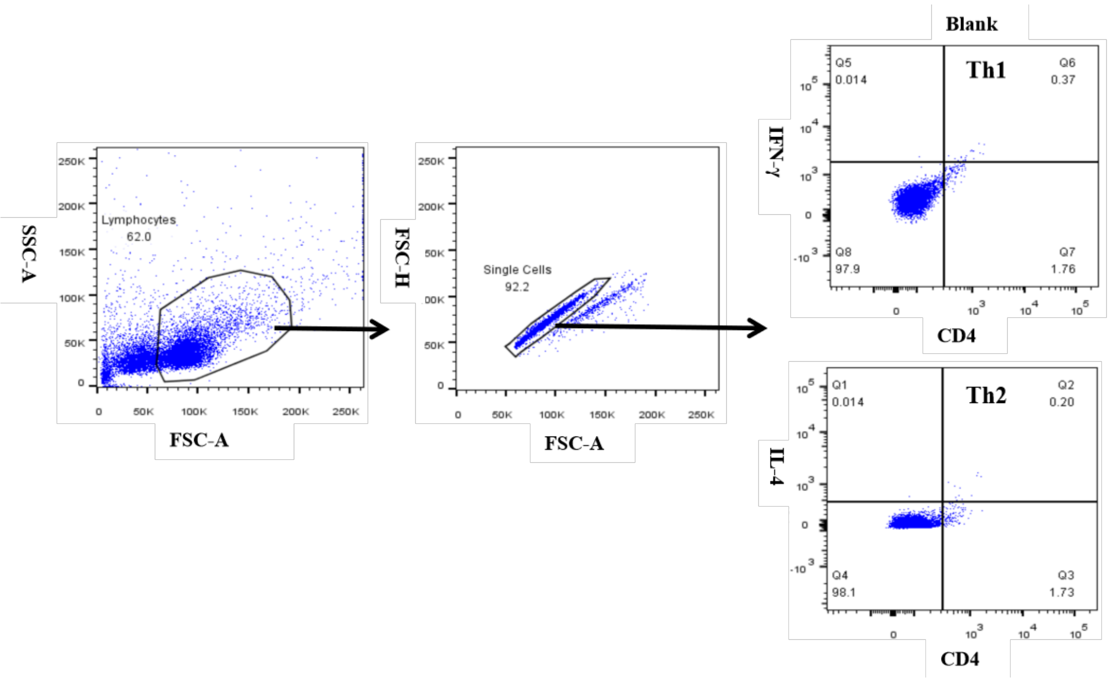

B

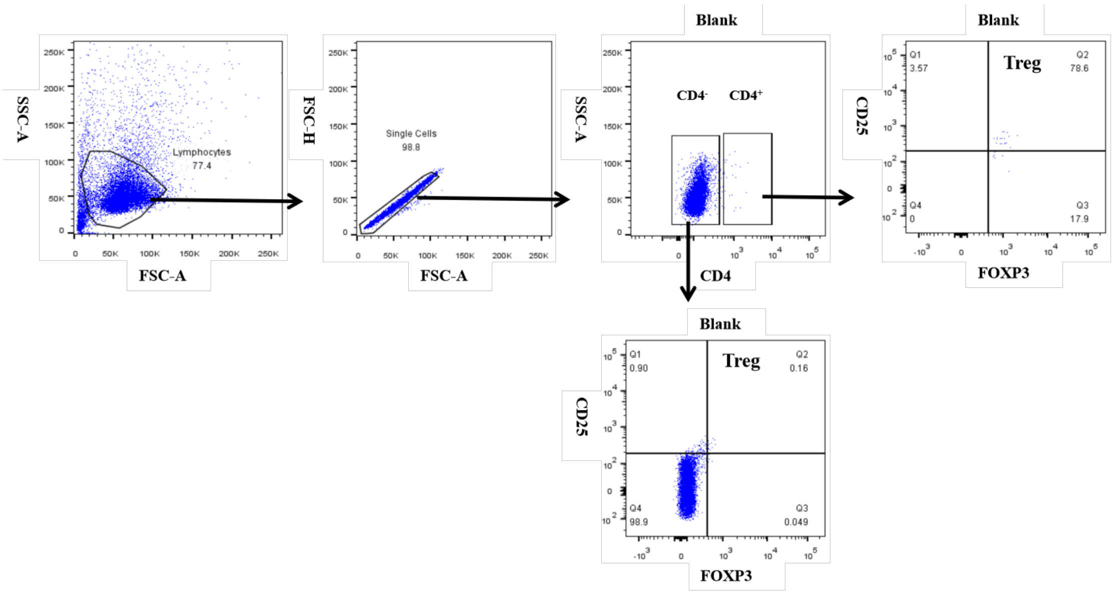

C

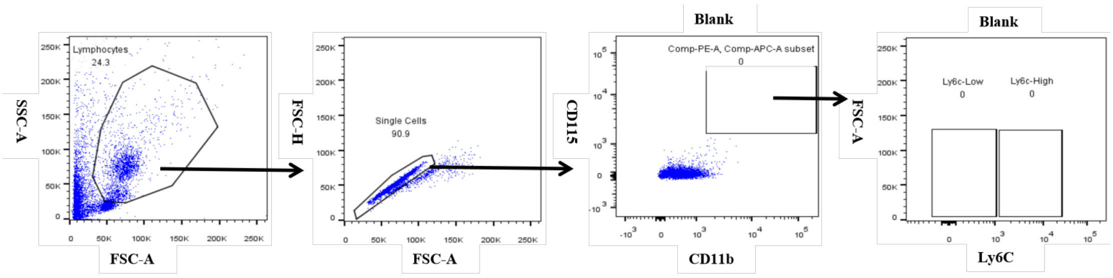

Figure S1. Blank controls for flow cytometry data from different cell types. (**A**) Blank control for Th cells in the spleen. (**B**)Blank control for Treg cells in the spleen. (**C**)Blank control for monocytes.

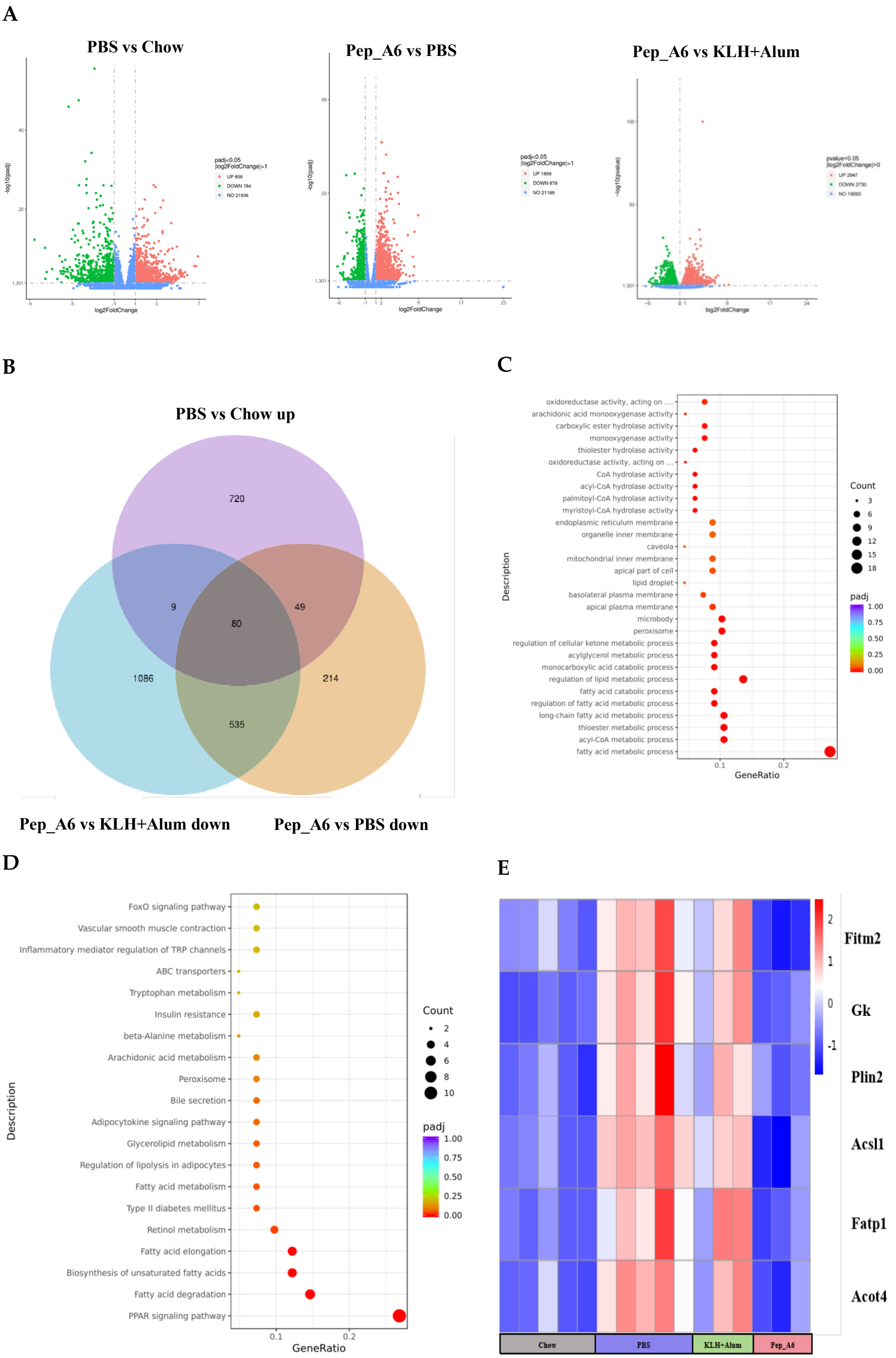
A
PBS vs Chow
Pep_A6 vs PBS
Pep_A6 vs KLH+Alum
log2FoldChange
-log10(padj)
-log10(pvalue)
padj<0.05
|log2FoldChange|>1
UP 858
DOWN 784
NO 21936
UP 1859
DOWN 878
NO 21169
pvalue<0.05
|log2FoldChange|>0
UP 2947
DOWN 2730
NO 18093
B
PBS vs Chow up
720
9
80
49
1086
214
535
Pep_A6 vs KLH+Alum down
Pep_A6 vs PBS down
C
oxidoreductase activity, acting on ....
arachidonic acid monooxygenase activity
carboxylic ester hydrolase activity
monooxygenase activity
thiolester hydrolase activity
oxidoreductase activity, acting on ...
CoA hydrolase activity
acyl-CoA hydrolase activity
palmitoyl-CoA hydrolase activity
myristoyl-CoA hydrolase activity
endoplasmic reticulum membrane
organelle inner membrane
caveola
mitochondrial inner membrane
apical part of cell
lipid droplet
basolateral plasma membrane
apical plasma membrane
microbody
peroxisome
regulation of cellular ketone metabolic process
acylglycerol metabolic process
monocarboxylic acid catabolic process
regulation of lipid metabolic process
fatty acid catabolic process
regulation of fatty acid metabolic process
long-chain fatty acid metabolic process
thioester metabolic process
acyl-CoA metabolic process
fatty acid metabolic process
Description
GeneRatio
0.1
0.2
Count
3
6
9
12
15
18
padj
1.00
0.75
0.50
0.25
0.00
D
FoxO signaling pathway
Vascular smooth muscle contraction
Inflammatory mediator regulation of TRP channels
ABC transporters
Tryptophan metabolism
Insulin resistance
beta-Alanine metabolism
Arachidonic acid metabolism
Peroxisome
Bile secretion
Adipocytokine signaling pathway
Glycerolipid metabolism
Regulation of lipolysis in adipocytes
Fatty acid metabolism
Type II diabetes mellitus
Retinol metabolism
Fatty acid elongation
Biosynthesis of unsaturated fatty acids
Fatty acid degradation
PPAR signaling pathway
Description
GeneRatio
0.1
0.2
Count
2
4
6
8
10
padj
1.00
0.75
0.50
0.25
0.00
E
Fitm2
Gk
Plin2
Acsl1
Fatp1
Acot4
2
1
0
-1
Chow
PBS
KLH+Alum
Pep_A6

F

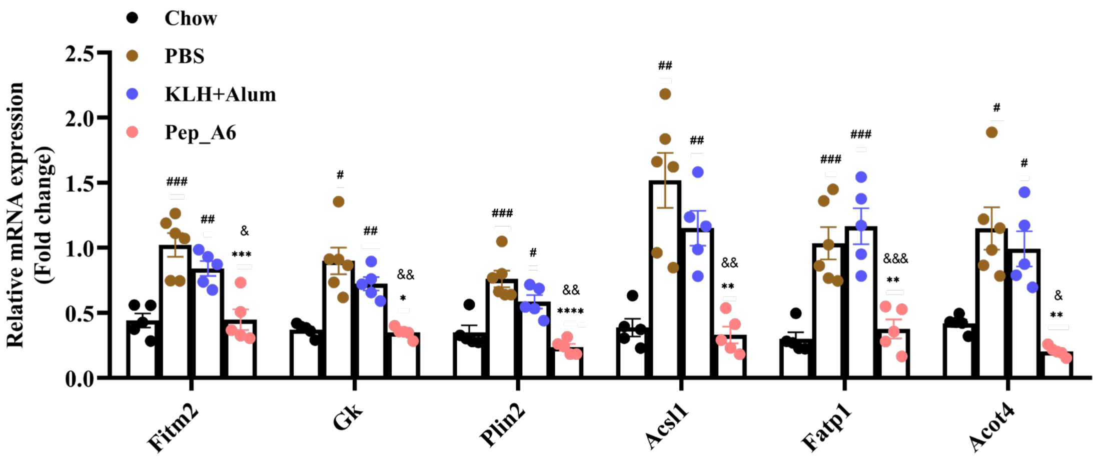

Figure S2. Pep_A6 Vaccination partially restored the expression of liver lipid metabolism-related genes up-regulated by a high-fat diet. (**A**) Volcano plots depicting differentially expressed genes (DEGs) in the PBS group compared to the Chow group, in the Pep_A6 vaccine group compared to the PBS group, and in the Pep_A6 vaccine group compared to the KLH+Alum group (n = 3-5, padj < 0.05). The red dots indicate gene up-regulation, and the green dots indicate gene down-regulation. (**B**) Venn plot showing overlapping genes between up-regulated from the HFD mice injected with PBS relative to the Chow mice, down-regulated from the Pep_A6-immunized mice relative to the HFD mice injected with PBS and down-regulated from the Pep_A6-immunized mice relative to the HFD mice injected with KLH+Alum mixture . Purple represents the up-regulated differential genes in the PBS group compared with Chow group, orange represents the down-regulated differential genes in the vaccine group compared with PBS group, light blue represents the down-regulated differential genes in the vaccine group compared with KLH+Alum group, and the gray region in the middle represents the overlapping DEGs among the three comparison combinations. (**C**)Bubble plot of GO enrichment analysis of overlapping DEGs down-regulated in vaccine groups (padj < 0.05); the bubble color from red to purple in the plot indicates the significance degree of gene enrichment(**D**) Bubble plot of KEGG enrichment analysis of overlapping DEGs (padj < 0.05); the bubble color from red to purple in the plot indicates the significance degree of gene enrichment. (**E**) Heatmaps displaying top-ranked genes related to lipid metabolism among the 80 overlapping DEGs. Red indicates gene upregulation, while blue indicates gene downregulation.(**F**) Quantitative polymerase chain reaction (qPCR) expression analysis of *Fitm2*, *Gk*, *Plin2*, *Acsl1*, *Fatp1* and *Acot4*. (Chow: n = 5; PBS: n = 6; KLH+Alum: n = 5; Pep_A6: n = 5. Data are expressed as mean ± SEM. $^{\#}P < 0.05$, $^{\#\#}P < 0.01$, $^{\#\#\#}P < 0.001$, as compared with the Chow group; $^{*}P < 0.05$, $^{**}P < 0.01$, $^{***}P < 0.001$, $^{****}P < 0.0001$, as compared with the PBS group; $^{\&}P < 0.05$, $^{\&\&}P < 0.01$, $^{\&\&\&}P < 0.001$, as compared with the KLH+Alum group)
